# Injuries in professional male football players in Kosovo: a descriptive epidemiological study

**DOI:** 10.1186/s12891-016-1202-9

**Published:** 2016-08-12

**Authors:** I. Shalaj, F. Tishukaj, N. Bachl, H. Tschan, B. Wessner, R. Csapo

**Affiliations:** 1 Institute of Sport Science, University of Vienna, Auf der Schmelz 6, A-1150 Vienna, Austria; 2 Austrian Institute of Sports Medicine, Auf der Schmelz 6, A-1150 Vienna, Austria; 3 Department of Sport Science, University of Innsbruck, Fürstenweg 187, A-6020 Innsbruck, Austria

**Keywords:** Soccer, Injury incidence, Epidemiology, Athlete, Trauma

## Abstract

**Background:**

The incidence and severity of football-related injuries has been found to differ strongly between professional leagues from different countries. The aims of this study were to record the incidence, type and severity of injuries in Kosovarian football players and investigate the relationship between injury incidence rates (IRs), players’ age and playing positions.

**Methods:**

Players’ age, anthropometric characteristics and playing positions, training and match exposure as well as injury occurrences were monitored in 11 teams (143 players) of Kosovo’s top division during the 2013/14 season. The exact type, severity and duration of football-related injuries were documented following International Federation of Football Associations (FIFA) recommendations.

**Results:**

A total of 272 injuries were observed, with traumatic injuries accounting for 71 %. The overall injury IR was 7.38 (CI: 7.14, 7.63) injuries per 1,000 exposure hours and ~11x lower during training as opposed to matches. Strains and ruptures of thigh muscles, ligamentous injuries of the knee as well as meniscus or other cartilage tears represented the most frequent differential diagnoses. While no statistical differences were found between players engaged in different playing positions, injury IR was found to be higher by 10–13 % in younger (IR = 7.63; CI: 7.39, 7.87) as compared to middle-aged (IR = 6.95; CI: 6.41, 7.54) and older players (IR = 6.76; CI: 5.71, 8.00).

**Conclusions:**

The total injury IR in elite football in Kosovo is slightly lower than the international average, which may be related to lesser match exposure. Typical injury patterns agree well with previously reported data. Our finding that injury IR was greater in younger players is related to a higher rate of traumatic injuries and may indicate a more aggressive and risky style of play in this age group.

**Electronic supplementary material:**

The online version of this article (doi:10.1186/s12891-016-1202-9) contains supplementary material, which is available to authorized users.

## Background

Football is a complex sporting activity, placing great demands on agility and involving most different movement patterns, such as jumps, sprints or side cut manoeuvres, that are frequently performed at high to maximal intensity [1]. The substantial physiological demands and the body contact between players account for the generally high injury incidence in this sport [2]. In recent years, several epidemiological studies have been performed to investigate the incidence and characteristics of injuries in professional football in a number of European and United States American leagues [3–6]. These studies revealed remarkable heterogeneity of injury incidences, as quantified by the number of injuries per 1,000 h of either training or match exposure. Particularly large differences were reported for injuries occurring during training, with numbers ranging from 2.9 in the United States American Major League Football [5] to 11.8 in the Danish Super League [4]. Also, injury patterns were found to differ between regions, with overall injury incidence being higher in northern European leagues, but specifically ligamentous injuries being observed more frequently in Mediterranean countries [7]. The reasons for these discrepancies between countries are likely multifactorial and may encompass differences in the total amount of training exposure and the periodisation of the competitive season [4], the number of matches played per season affecting recovery times between competitions [8, 9], and regional differences in the style of play [10, 11]. Ultimately, knowledge about and application of appropriate injury-prevention programmes as well as the availability and quality of medical care may also strongly influence the incidence of football-related injuries [12]. Taken together, these results demonstrate the inherent difficulties in extrapolating epidemiological data obtained in one country to other leagues or playing environments. Therefore, the completion of regional epidemiological studies to characterise country-specific injury profiles is of utmost importance.

To date, no studies have investigated football-related training and match exposure as well as injuries in Kosovo. The Raiffeisen Football Super League differs from bigger European leagues in that Kosovo has only very recently been admitted as a member of both the International Federation of Football Associations (FIFA) and the Union of European Football Associations (UEFA). Hence, at the time this study was conducted, its teams did not yet compete at the international level, which might result in lesser match exposure of players and, possibly, a lower level of professionalism as compared to other countries. These particularities render the study of football-related injuries in Kosovo a particularly worthwhile goal.

Furthermore, it is of special interest to investigate whether the types and incidence rates of football-related injuries would be influenced by specific risk factors, such as players’ age or playing position. As regards the possible association of age with injury rates, conflicting evidence has been reported in the literature [13–15]. Further, it is not currently known whether younger and older players would be predominantly affected by certain types of injuries. In this regard, one could speculate that a potentially more aggressive and risky style of play would coincide with a greater ratio of traumatic injuries in younger players, whereas overuse syndromes would occur more frequently at older age. Further, injury risk has been found to be greatest in goal-near zones of the pitch [16], which suggests that injury rates would have to be greater in defenders and forwards as compared to midfielders. However, studies to investigate the relationship between playing position and injury rates have shown equivocal results [5, 17–21].

In the light of these considerations, the present study aimed to (i) prospectively record the incidence, type and severity of injuries in elite Kosovarian football players over a complete season; and (ii) investigate the relationship between injury incidence, players’ age and playing positions.

## Methods

### Study cohort

All 12 teams participating in the Raiffeisen Football Superleague (the top division of football in Kosovo) with a total number of 263 players were invited to contribute to this investigation. Eleven teams and a total of 143 male players gave their written informed consent to participate in the study. Complete data sets could be acquired in all players included.

### Data acquisition procedures

The data acquisition was carried out during the autumn and spring season of 2013/2014. Following the acquisition of players’ baseline characteristics (including anthropometric data, playing position as well the history of previous injuries) during preseason, training and match exposure times were documented by a member of the teams’ medical staff (team physician or physiotherapist) for each individual player, and submitted to the principal investigator on a weekly basis. The same medical expert was also responsible for the assessment of the exact type, severity and duration of football-related injuries, as well as the circumstances under which they occurred. For this purpose, specific injury report forms were used [22]. All injuries resulting in a player being unable to fully engage in training or match play were recorded, and followed up until return to play was recommended by the team physician. Injuries necessitating absence from play between 1–3, 4–7, 8–28 and more than 28 days were classified as minimal, mild, moderate and severe, respectively [22]. Teams were provided with a study manual containing detailed information on how to record data, including explanatory examples. To verify appropriate data acquisition, reports were routinely checked by the principal investigator on a weekly basis, and feedback was provided to teams to correct missing or unclear data. The study followed the recommendations outlined in the FIFA Medical Assessment and Research Centre (F-MARC) consensus on definitions and data collection procedures in studies of football injuries, and used translated versions of the according forms provided by Fuller and colleagues [22].

### Data analyses

Data are presented as means ± standard deviations (SD) and absolute or relative frequencies. To compare the likeliness of injuries between matches and training as well as autumn and spring season, injury incidence rates (IR) per 1,000 exposure hours (as documented on the player level) and associated 95 % CIs were calculated using Poisson regressions with generalized estimation equations. This approach allows to account for the cluster effect of teams [23] and has, in recent years, increasingly been used in epidemiological studies into sports-related injuries [24–26].

To assess the influence of age, IRs were independently calculated for players assigned to groups of young (≤24 years; *n* = 93), middle (24–29 years; *n* = 29) or older (> 29 years; *n* = 14) age. For the comparison of injury IRs across playing positions, age was included into the Poisson regression as continuous covariate variable, to control for potential bias related to age differences between defenders, midfielders and forwards [13]. Further, to increase statistical power, defenders (internal and external, *n* = 47), midfielders (central and external, *n* = 41) and forwards (wingers and strikers, *n* = 48) were pooled for comparisons of IRs across playing positions. Considering the low numbers of players (*n* = 7) and injuries suffered (*n* = 11), keepers were excluded from these analyses. All statistical analyses were carried out using the SPSS statistical software package (SPSS 23.0, IBM Corp., Armonk, NY).

## Results

### Player baseline characteristics

The total sample of 143 players was composed of 7 goalkeepers (4.9 %), 27 internal (18.9 %) and 20 external (14.0 %) defenders, 18 central (12.6 %) and 23 external (16.1 %) midfielders as well as 20 wingers (14.0 %) and 28 strikers (19.6 %). The players’ age, body mass, height and body mass index (BMI) were 23.2 ± 4.1 years, 74.2 ± 6.7 kg, 180.0 ± 5.3 cm and 22.9 ± 1.7 kg∙m^−2^, respectively. The dominant leg, defined as the leg preferentially used to kick the ball, was the right in 129 (90.2 %) and the left in 14 (9.8 %) players.

### Training and match exposure

Over the entire study period, a total of 36,833 h of exposure, consisting of 31,998 h of training and 4,834 h of match play, were registered. On average, players participated in 25.3 ± 4.0 matches and attended to 149.2 ± 14.3 training sessions. This resulted in a mean exposure time of 257.6 ± 24.9 h, including 33.8 ± 8.9 h (13.1 %) of match play and 223.8 ± 21.5 h (86.9 %) of training per player.

### Overall injury incidence rate

A total of 272 injuries, out of which 135 (49.6 %) occurred during the autumn and 137 (50.4 %) during the spring season, were recorded over the observation period. Normalisation to total exposure time yielded an IR of 7.38 (CI: 7.14, 7.63) injuries per 1,000 exposure hours. On average, each player sustained 1.9 injuries over the course of the competitive season. Injury IRs were substantially higher during competitions (*n* = 171; IR = 35.37; CI: 31.96, 39.14) as compared to trainings (*n* = 101; IR = 3.16; CI: 2.70, 3.69). Calculation of the respective IRR revealed a ~11x greater injury rate during matches as compared to trainings (IRR = 11.21; CI: 8.76, 14.33; *P* < 0.001). Similar absolute numbers of injuries were recorded in the autumn (*n* = 135) and spring (*n* = 137) season. Normalisation to exposure time revealed that injury IR was slightly higher in the spring (IR = 8.08; CI: 7.75, 8.43) as compared to the autumn season (IR = 6.79; CI: 6.46, 7.14). However, calculation of the respective IRR revealed that this difference in injury IR between seasons was not statistically significantly (IRR = 1.19; CI: 0.94, 1.51; P = 0.153).

### Traumatic injuries vs. overuse syndromes

**Fig. 1:**
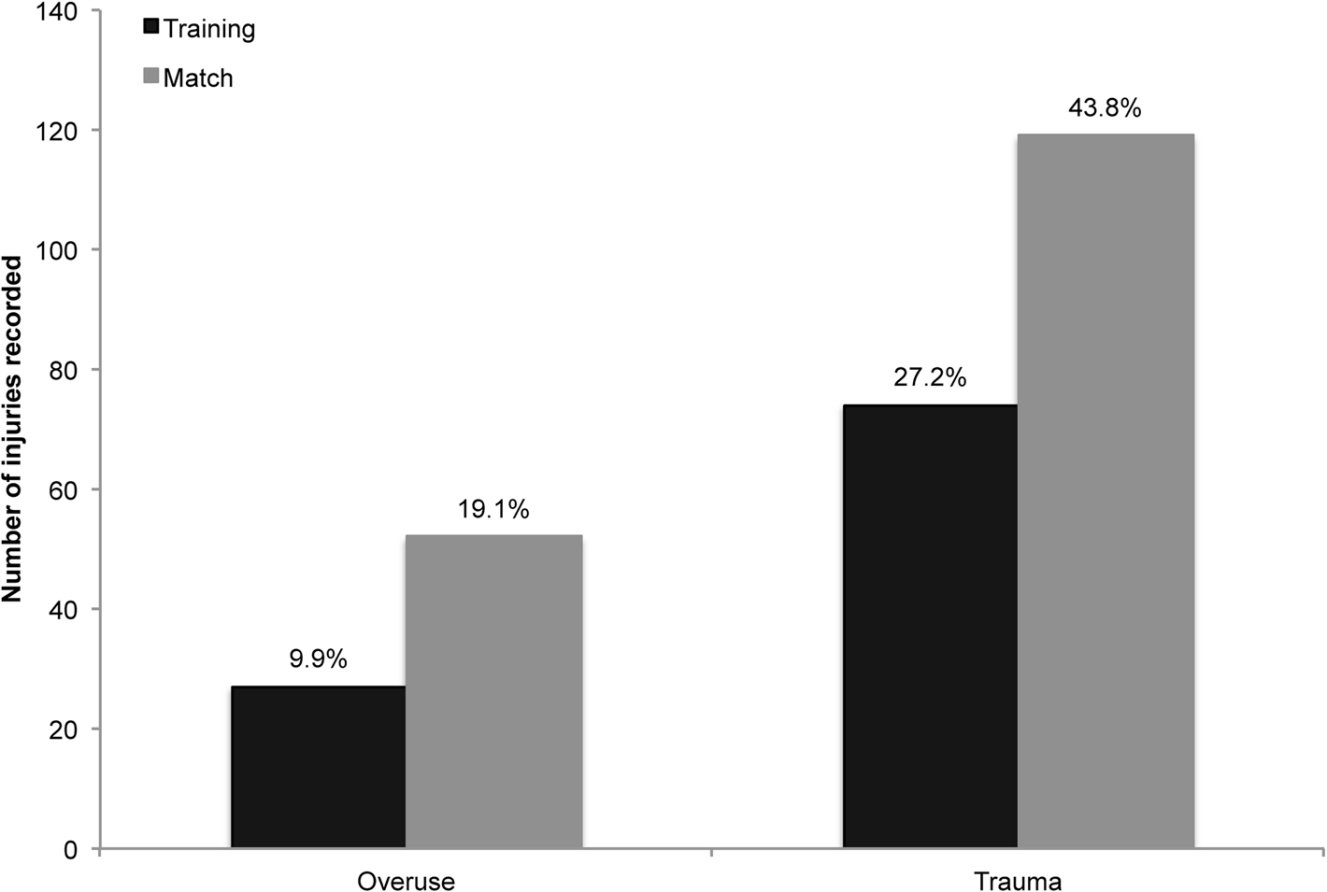
Relative numbers of overuse and traumatic injuries occurring during matches and training

The breakdown of injuries into traumatic and overuse injuries (see Fig. 1) showed that traumatic injuries comprised 70.96 % of all injuries (*n* = 193). Normalisation to exposure times revealed IRs of 5.24 (CI: 4.84, 5.68) and 2.15 (CI: 1.77, 2.60) for traumatic and overuse injuries, respectively. The respective IRR was 2.44 (CI: 1.88, 3.17), reflecting a significantly higher rate of traumatic injuries (*P* < 0.001). Of note, IRRs showed that rates of both traumatic (IRR = 10.64; CI: 7.96, 14.23; *P* < 0.001) and overuse injuries (IRR = 12.75; CI: 8.01, 20.29; *P* < 0.001) were more than 10x greater in matches as compared to training.

### Injury location and severity

While 72.0 % of all injuries affected the lower extremities, the knee, thigh and ankle were the single body parts most commonly affected by injury, jointly accounting for 47.8 % of all injuries recorded. Also, injuries to these body regions represented the largest groups of injuries classified as moderate (resulting in absence of play between 8 and 28 days, 56.5 %) or severe (absence > 28 days, 63.0 %). Table 1 summarizes the breakdown of injuries by location and severity. As regards the types of injuries, muscle strains, ligament injuries and contusions were most frequently reported. Taken together, these injury types accounted for 61.4 % of all lesions recorded. The breakdown of injuries by type and severity is evident from Table 2. Strains or ruptures of thigh muscles represented the single most common form of injury, with a total of 39 (14.3 %) observed cases. Further frequently reported differential diagnoses were ligamentous injuries of the knee joint (*n* = 33, 12.1 %), meniscus or other cartilage tears (*n* = 17, 6.3 %) as well as calf muscle strains or ruptures (*n* = 15, 5.5 %).

**Table 1:** Injuries by location and severity

Injury location	1–3 days		4–7 days		8–28 days		>28 days		Total	
Knee	1	(0.4 %)	20	(7.4 %)	35	(12.9 %)	4	(1.5 %)	60	(22.1 %)
Thigh	8	(2.9 %)	11	(4.0 %)	14	(5.1 %)	11	(4.0 %)	44	(16.2 %)
Ankle	3	(1.1 %)	9	(3.3 %)	12	(4.4 %)	2	(0.7 %)	26	(9.6 %)
Shank/Achilles tendon	8	(2.9 %)	8	(2.9 %)	8	(2.9 %)	-	(0.0 %)	24	(8.8 %)
Hip/groin	6	(2.2 %)	9	(3.3 %)	7	(2.6 %)	-	(0.0 %)	22	(8.1 %)
Foot/toe	5	(1.8 %)	7	(2.6 %)	5	(1.8 %)	1	(0.4 %)	18	(6.6 %)
Shoulder/clavicles	-	(0.0 %)	2	(0.7 %)	7	(2.6 %)	2	(0.7 %)	11	(4.0 %)
Elbow	-	(0.0 %)	3	(1.1 %)	6	(2.2 %)	2	(0.7 %)	11	(4.0 %)
Forearm	2	(0.7 %)	4	(1.5 %)	3	(1.1 %)	2	(0.7 %)	11	(4.0 %)
Lower back/pelvis	3	(1.1 %)	4	(1.5 %)	1	(0.4 %)	-	(0.0 %)	8	(2.9 %)
Thorax/upper back	1	(0.4 %)	3	(1.1 %)	1	(0.4 %)	2	(0.7 %)	7	(2.6 %)
Wrist	-	(0.0 %)	2	(0.7 %)	4	(1.5 %)	1	(0.4 %)	7	(2.6 %)
Upper arm	2	(0.7 %)	3	(1.1 %)	1	(0.4 %)	-	(0.0 %)	6	(2.2 %)
Head/face	1	(0.4 %)	4	(1.5 %)		(0.0 %)	-	(0.0 %)	5	(1.8 %)
Hand/finger/thumb	1	(0.4 %)	1	(0.4 %)	3	(1.1 %)	-	(0.0 %)	5	(1.8 %)
Abdomen	3	(1.1 %)	-	(0.0 %)	1	(0.4 %)	-	(0.0 %)	4	(1.5 %)
Neck/cervical spine	-	(0.0 %)	3	(1.1 %)	-	(0.0 %)	-	(0.0 %)	3	(1.1 %)
Total	44	(16.2 %)	93	(34.2 %)	108	(39.7 %)	27	(9.9 %)	272	(100.0)%

*Note:* Values in parentheses represent % of total (272) injuries

**Table 2:** Injuries by type and severity

Injury type	1–3 days		4–7 days		8–28 days		>28 days		Total	
Muscle injury/strain	15	(5.5 %)	20	(7.4 %)	20	(7.4 %)	10	(3.7 %)	65	(23.9 %)
Sprain/ligament injury	-	(0.0 %)	29	(10.7 %)	28	(10.3 %)	-	(0.0 %)	57	(21.0 %)
Contusion	-	(0.0 %)	24	(8.8 %)	20	(7.4 %)	1	(0.4 %)	45	(16.5 %)
Meniscus/cartilage	-	(0.0 %)	-	(0.0 %)	18	(6.6 %)	4	(1.5 %)	22	(8.1 %)
Abrasion	17	(6.3 %)	3	(1.1 %)	-	(0.0 %)	-	(0.0 %)	20	(7.4 %)
Dis- or subluxation	-	(0.0 %)	-	(0.0 %)	9	(3.3 %)	8	(2.9 %)	17	(6.3 %)
Tendon injury	-	(0.0 %)	6	(2.2 %)	7	(2.6 %)	-	(0.0 %)	13	(4.8 %)
Bruise	10	(3.7 %)	3	(1.1 %)	-	(0.0 %)	-	(0.0 %)	13	(4.8 %)
Fracture	1	(0.4 %)	-	(0.0 %)	2	(0.7 %)	4	(1.5 %)	7	(2.6 %)
Laceration	-	(0.0 %)	4	(1.5 %)	3	(1.1 %)	-	(0.0 %)	7	(2.6 %)
Concussion	-	(0.0 %)	4	(1.5 %)	-	(0.0 %)	-	(0.0 %)	4	(1.5 %)
Other bone injury	-	(0.0 %)	-	(0.0 %)	1	(0.4 %)	-	(0.0 %)	1	(0.4 %)
Dental injury	1	(0.4 %)	-	(0.0 %)	-	(0.0 %)	-	(0.0 %)	1	(0.4 %)
Nerve injury	-	(0.0 %)	-	(0.0 %)	-	(0.0 %)	-	(0.0 %)	-	(0.0 %)
Total	44	(16.2 %)	93	(34.2 %)	108	(39.7 %)	27	(9.9 %)	272	(100.0 %)

*Note:* Values in parentheses represent % of total (272) injuries

The largest portion of all recorded injuries were classified as moderate, thus requiring an absence from play between 8 and 28 days (*n* = 108, 39.7 % of all injuries). Here, the most common subtypes of injuries were joint sprains or ligament injuries (*n* = 28, 10.3 %), followed by muscle strains and contusions (both *n* = 20, 7.4 %). The body parts most frequently affected by moderate injuries were the knee (*n* = 35, 12.9 %), thigh (*n* = 14, 5.1 %) and ankle (*n* = 12, 4.1 %). Relatively fewer injuries were classified as mild (4–7 days of absence; *n* = 93, 34.2 %) and minimal (1–3 days of absence; *n* = 44, 16.2 %). Only 27 injuries (9.9 %) were severe enough to require an absence from play of more than 28 days.

### Injuries in dependency of playing position

**Fig. 2:**
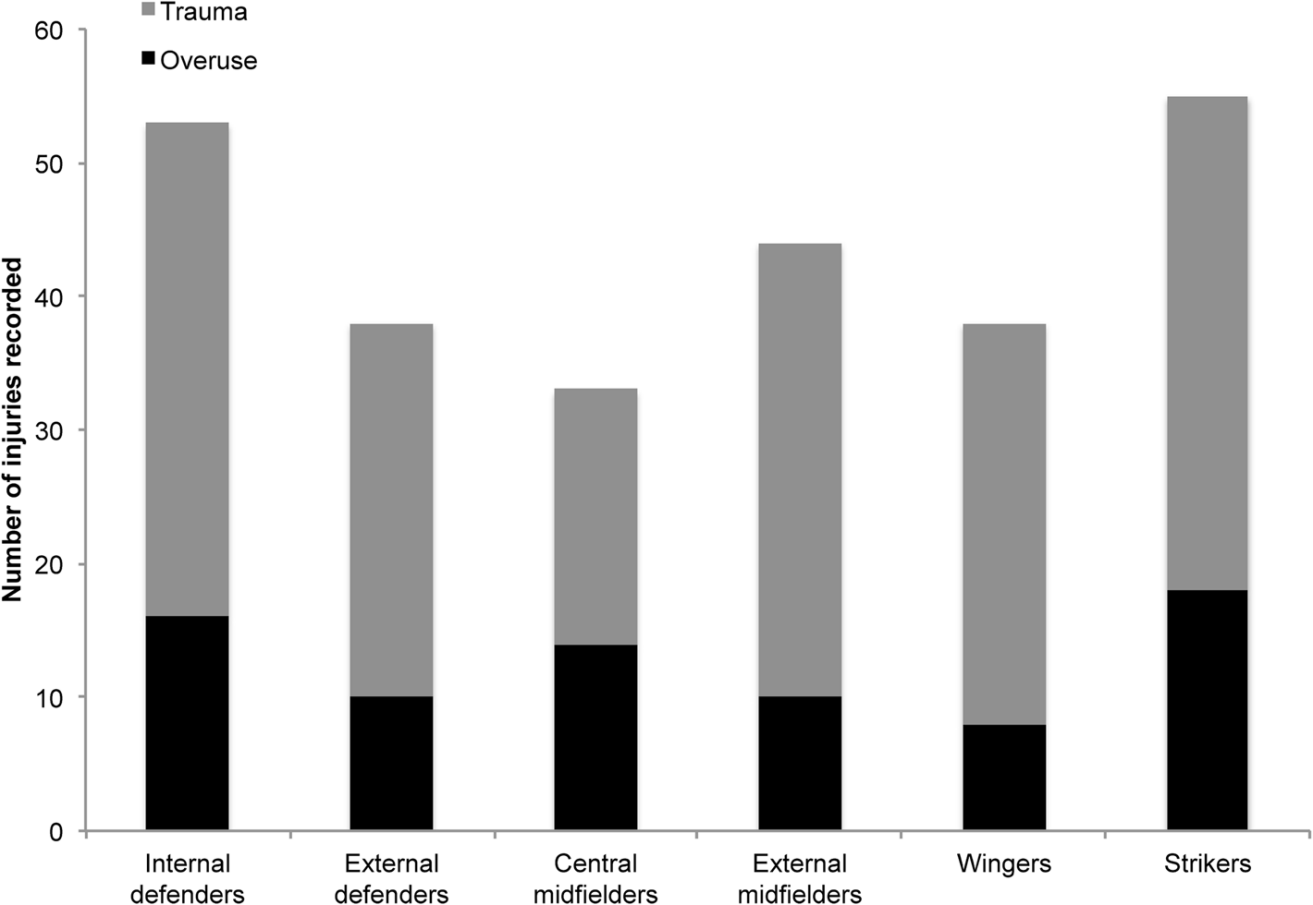
Numbers of overuse and traumatic injuries in dependency of playing position

Considering the low number of injuries suffered (*n* = 11), goalkeepers were excluded from the statistical analyses of injuries in dependency of playing position. For outfield players, a total of 261 injuries were recorded. In absolute terms, strikers (*n* = 55, 21.1 % of all injuries) and internal defenders (*n* = 53, 20.3 %) represented the groups most frequently affected, followed by external midfielders (*n* = 44, 16.9 %), external defenders and wingers (both *n* = 38, 14.6 %) as well as central midfielders (*n* = 33, 12.6 %) (Fig. 2).

For comparison of injury IRs, defenders (internal and external, *n* = 47), midfielders (central and external, *n* = 41) and forwards (wingers and strikers, *n* = 48) were pooled for statistical analysis. On average, defenders represented the youngest group (22.6 ± 4.1 years), whereas midfielders were oldest (23.5 ± 4.1 years). While differences in age were not statistically significant (F(2,133) = 0.767, P = 0.467), age was still considered as covariate in the Poisson regression analyses to investigate potential age-related bias (see Methods section under *Data analyses*). The respective injury IRs were 7.74 (CI: 7.36, 8.13), 7.30 (CI: 6.79, 7.84) and 7.32 (CI: 7.00, 7.66) for defenders, midfielders and forwards, respectively. Calculation of IRRs revealed that injury rates did neither differ significantly between defenders and midfielders (IRR = 1.05; CI: 0.97, 1.14; *P* = 0.250), defenders and forwards (IRR = 1.05; CI: 1.00, 1.13; *P* = 0.124) nor midfielders and forwards (IRR = 1.00; CI: 0.82, 1.08; *P* = 0.968). Also, the effect of age was weak for all comparisons, with annual changes in IR being smaller than 2 % (Defenders vs. midfielders: Exp(B) = 0.99; CI: 0.98, 1.01; *P* = 0.214. Defenders vs. forwards: Exp(B) = 1.00; CI: 0.99, 1.01; *P* = 0.574. Midfielders vs. forwards: Exp(B) = 0.99 (CI: 0.98, 1.00, *P* = 0.026).

### Injuries in dependency of age

To assess whether age affected injury risk, players were categorised as either young (≤ 24 years; *n* = 97), middle (25–29 years; *n* = 31) or old (> 29 years; *n* = 15). The total numbers of injuries recorded in the young, middle and older group were 189, 57 and 26, respectively. Normalisation to exposure times revealed that injury IR was slightly higher in the young (IR = 7.63; CI: 7.39, 7.87) as compared to the middle (IR = 6.95; CI: 6.41, 7.54) and older age group (IR = 6.76; CI: 5.71, 8.00). Calculation of IRRs demonstrated that these differences were statistically significant for young and middle-aged players (IRR = 1.10; CI: 1.01, 1.20; *P* = 0.038) but not for comparisons between other groups (Young vs. old: IRR = 1.13; CI: 0.95, 1.34; *P* = 0.168. Middle vs. old: IRR = 1.03; CI: 0.85, 1.24; *P* = 0.769).

Within young players, 142 injuries (75.1 % of injuries in this age group) were classified as traumatic and 47 (24.9 %) as overuse syndromes. In the middle-aged and older groups, by contrast, the ratio of traumatic injuries was somewhat lower. Here, only 35 (61.4 %) and 16 (61.5 %) traumatic injuries, respectively, were recorded. Normalisation to exposure times confirmed the notion that younger players were slightly more frequently affected by traumatic injuries: Injury IRs were lower in the middle-aged (IR = 4.27; CI: 3.43, 5.32) and older group (IR = 4.16; CI: 3.14, 5.51), as compared to the young cohort (IR = 5.73; CI: 5.27, 6.23). IRRs demonstrated that the rates of traumatic injuries were significantly higher in young players as compared to both other age groups (Young vs. middle: IRR = 1.34; CI: 1.06, 1.70; *P* = 0.014. Young vs. old: IRR = 1.38; CI: 1.03, 1.85; *P* = 0.032). Between middle-aged and older players, by contrast, no statistical differences were found (IRR = 1.03; CI: 0.89, 1.03; *P* = 0.887).

## Discussion

The present study represents the first epidemiological investigation into football-related injuries in Kosovo. Monitoring nearly 37,000 exposure hours in 11 clubs and 143 players from the Raiffeisen Football Superleague, a total of 272 injuries were observed. These findings coincide with an overall injury IR of 7.4 (CI: 7.1, 7.6) injuries per 1000 exposure hours. Approximately 3 out of 4 injuries were classified as either moderate (39.7 %) or mild (34.2 %), whereas only 1 out of 10 injuries (9.9 %) entailed an absence of play of more than 28 days.

### Overall injury incidence rate

Studies to investigate football-related injuries have been performed in various major football leagues. These studies have evidenced considerable heterogeneity in the incidence of injuries between countries. The overall injury IR observed in this study for the Kosovo Football Superleague (IR = 7.4; CI: 7.1, 7.6) was ~20 % below the international average (Table 3). One parameter that has been associated with injury incidence is the amount of exposure and, particularly, the total number of matches played over one season since this factor directly affects recovery times between competitions [8, 9]. In our study, players engaged in 25.3 matches on average, which is equivalent to 33.8 h of match exposure. Comparison with previously published studies demonstrates that players engaged in bigger football leagues are subject to a substantially greater number of matches. For instance, top teams in northern and southern Europe were found to face 32.3 (+22 %) and 34.8 (+28 %) matches a year, respectively [7]. While top teams may resort to larger numbers of elite players and can therefore rotate their squads more frequently (differences in average match hours were only 14 % and 18 %, respectively), the significantly lower match exposure may explain the smaller overall injury incidence in Kosovo. The reasons for the lower number of matches played per season may be related to Kosovo not yet being a member of either FIFA or UEFA and its teams, therefore, not competing at the international level at the time this study was conducted. Training times, by contrast, agreed well with data reported from top teams in southern Europe (+2 %) and were even slightly greater than those observed in northern Europe (+9 %) [7].

**Table 3:** Incidence rates (95 % confidence intervals) of football injuries in different countries

Country/Origin	Study	Total	Training	Match
Denmark^a^	Hagglund et al. [4]	14.4 (9.1, 19.8)	11.8 (6.7, 16.9)	28.2 (17.8, 38.7)
Iceland	Arnason et al. [30]	12.4 (12.1, 12.7)	5.9 (5.7, 6.1)	34.8 (33.6, 36.0)
UEFA CL	Walden et al. [32]	9.4 (9.2, 9.6)	5.8 (5.6, 6.0)	30.5 (29.4, 31.6)
Sweden^a^	Hagglund et al. [4]	8.2 (5.5, 11.0)	6.0 (3.9, 8.2)	26.2 (16.8, 35.5)
Sweden	Hagglund et al. [14]	7.6 (7.1, 8.3)	5.1 (4.6, 5.6)	25.9 (22.8, 29.2)
Kosovo^a^	Present	7.4 (7.1, 7.6)	3.2 (2.7, 3.7)	35.4 (32.0, 39.1)
USA	Morgan et al. [5]	6.2 (n/a)	2.9 (n/a)	35.3 (n/a)
Netherlands^a^	Stubbe et al. [31]	6.2 (5.5, 7.0)	2.8 (2.3, 3.3)	32.8 (28.2, 38.1)
Average ± SD	-	9.0 ± 2.8	5.4 ± 2.7	31.1 ± 3.7

*Note:* Incidence rates are reported as injuries per 1000 exposure hours. This list is not exhaustive. Data acquisition in studies denoted with ^a ^has been performed in agreement with the consensus on definitions and data collection procedures in studies of football injuries [22]. *UEFA CL* Union of European Football Associations Champions League. Average ± SD reflect the means and standard deviations of the incidence rates reported in the studies included in this table

Yet another explanation for the relatively low injury incidence in Kosovo may lie in the “northern bias” described by Walden and colleagues [7]. These authors reported that injury incidence was generally higher among teams from regions in northern Europe (that have also been more extensively studied) as compared to teams from regions with a Mediterranean climate type. This may be due to differences in environmental playing conditions. A study performed in Scottish rugby players concluded that greater wind strength and lower external temperatures contributed to increased risk of injury [27]. Hence, it may be speculated that the comparably warm environmental conditions during the football season in Kosovo would protect players against certain types of non-contact (e.g. muscle strain) injuries. Ultimately, regional differences in injury incidence might also be related to particularities in the style of play. For instance, Dellal and colleagues [11] found that players in the English Premier League covered significantly greater distances sprinting than those engaged in the Spanish La Liga. While physical and technical aspects of match-play were not assessed in this study, it is possible that a more technical and physically less demanding style of play would be associated with lower overall injury risk.

### Injury types

In our study, the most frequent injury types were muscle strains, ligament injuries and contusions, which jointly accounted for more than 60 % of all injuries. Strains or ruptures of thigh muscles (*n* = 39; 14.3 %), ligamentous injuries of the knee joint (*n* = 33, 12.1 %), meniscus or other cartilage tears (*n* = 17, 6.3 %) as well as calf muscle strains or ruptures (*n* = 15, 5.5 %) represented the most frequent differential diagnoses. These findings are in agreement with the results of previous epidemiological studies conducted in football. For instance, in an extensive review paper by Wong and Hong [28] 21 of the studies included reported contusions and 6 strains as the most common injury type. Thigh muscle strains or ruptures, mostly affecting the posterior aspect of the thigh, represented the single most common differential diagnosis. In a longitudinal study, monitoring 27 teams over a period of 11 years, hamstring strains were similarly reported as the most common injury type, accounting for 12.8 % of all injuries [29]. This figure agrees well with the 14.3 % found in the present investigation. Also, our observation that the rate of injuries classified as traumatic (IR = 5.2; accounting for 71 % of all injuries) was ~2.4x greater than that of overuse syndromes (IR = 2.2) coincides reasonably well with the results of previous studies [2, 28] and underlines that the majority of injuries encountered in professional football are acute trauma.

### Injuries in match vs. training

Our data showed that injury rate was ~11x higher during matches (IR = 35.4) as compared to trainings (IR = 3.2). While this finding agrees well with previously published data [4, 5, 14, 30–32] (cf. Table 3), it is interesting to note that this ratio was similar for both traumatic (IRR = 10.64; CI: 7.96, 14.23; *P* < 0.001) and overuse injuries (IRR = 12.75; CI: 8.01, 20.29; *P* < 0.001). A study by Rahnama and colleagues [16], who investigated playing actions associated with increased injury risk in football matches, may help explain the drastically higher injury rates in matches. These authors reported that actions entailing a substantial injury risk involved, amongst others, tackles, kicks, headings and goal catches. It may be speculated that these actions occur more frequently and are performed with greater intensity during matches. Further, it has been found that injuries tend to occur more frequently toward the end of each half [2, 6, 33], suggesting a possible role of fatigue in the increased likeliness of injuries during matches.

### Injuries in dependency of age and playing position

Previous studies to investigate the association between playing position and injury rates in football have shown controversial results. While some studies reported that defenders were particularly prone to ankle sprain [18] and hamstring strain injuries [21], others found the greatest injury rates in forwards [20, 34]. Several further papers reported no association between injury rates and playing position [5, 17, 19]. One factor that may contribute to this heterogeneity of results is players’ age. While evidence is somewhat conflicting [14], some studies have suggested that injury rates in football would be increasing with age [13, 15]. In our study, injury IR was slightly higher in the young (IR = 7.6) as compared to the middle (IR = 7.0) and older (IR = 6.8) age group. These results reflect ~10–13 % higher rates of injuries in the youngest age group, with differences between the young and middle age group reaching statistical significance. Our analyses further suggested that the differences in overall IR were particularly due to unequal IRs of traumatic injuries that occurred at significantly higher rates in younger players (1.34 < IRR < 1.38; *P* < 0.05). These results might indicate a more aggressive and risky style of play in younger athletes.

Considering the significant differences between age groups, we decided to include age as covariate into Poisson regression analyses, to account for potential age-related bias when comparing injury IR between players engaged in different playing positions. However, inspection of the respective factor weights indicated no substantial bias related to age (0.99 < Exp(B) < 1.00). After pooling of players into groups of defenders, midfielders and forwards, our results showed that injury IR were slightly higher in defenders (IR = 7.7) as compared to midfielders and forwards (both: IR = 7.3). This observation may be related to the notion that, in matches (where the vast majority of injuries occurs), injury risk is greatest in those areas of the pitch where possession of the ball is most vigorously contested, such as the defending zones close to the goal [16]. However, it is important to note that differences were small and statistically not significant. Hence, our results support previous studies to suggest that the rates at which injuries occur are not dependent on playing position [5, 17, 19].

### Methodological considerations

The fact that the injury occurrences reported in this study relied strictly on the judgement of members of the teams’ medical staff must be recognised as potential limitation of our study. Differential diagnoses could not be verified by a single, supervisory expert. Further, the age distribution of players was skewed, with the preponderance of athletes being 24 years of age or younger (97/143) and comparably few being 29 years or older (15/143). We acknowledge that such skewness may introduce bias when comparing injury IRs across age groups. Finally, for comparisons between playing positions, players had to be pooled into groups of defenders, midfielders and forwards. It should be noted that video-based match analyses have demonstrated that players acting closer to the touchlines, such as external midfielders, perform a greater number of sprints and cover larger distances sprinting (potentially increasing the risk of strain injuries) than those operating near the centre of the pitch [35, 36]. Thus, while required to increase statistical power, the pooling of central and external players complicates the derivation of position-specific injury profiles.

## Conclusions

The overall incidence of injuries in football in Kosovo is slightly below the average of other European leagues. This result may be related to Kosovarian players engaging in substantially fewer matches than players of teams also competing at the international level. In agreement with the results of previous epidemiological studies in football, nearly 3 out of 4 injuries were classified as traumatic. Injury IR were more than 10-fold greater during matches as compared to trainings. Strains and ruptures of thigh muscles, ligamentous injuries of the knee as well as meniscus or other cartilage tears represented the most frequent differential diagnoses. While no differences were found between players engaged in different playing positions, injury IRs were found to be significantly greater in younger as compared to middle-aged and older players. This study represents the first ever epidemiological investigation into football injuries in Kosovo and, thus, provides novel information about the incidence, type and severity of injuries in an emerging European football league. Building up on these data, future studies will identify risk factors for the most prevalent differential diagnoses and aim to develop tailor-made prevention programmes.

## Additional file

Additional file 1: Raw Data. (XLSX 23 kb)

## Abbreviations

BMI: Body Mass Index
CIs: Confidence Intervals
FIFA: International Federation of Football Associations
F-MARC: FIFA Medical Assessment and Research Centre
IR: Injury Incidence Rate
IRR: Injury Incidence Rate Ratio
SD: Standard Deviation
UEFA: Union of European Football Associations
